# Fournier Gangrene Management: Is a Stoma Mandatory?

**DOI:** 10.7759/cureus.39450

**Published:** 2023-05-24

**Authors:** Margarida Dupont, Clara Leal, Nadia Tenreiro, Rita Marques, João Pinto-de-Sousa

**Affiliations:** 1 General Surgery, Centro Hospitalar de Trás-os-Montes e Alto Douro, Vila Real, PRT; 2 General Surgery, Centro Hospitalar de Trás-Os-Montes e Alto Douro, Vila Real, PRT

**Keywords:** necrotizing fasciitis, surgical debridement, stoma, negative pressure wound therapy, fournier gangrene

## Abstract

Fournier gangrene is a rare life-threatening surgical emergency mainly due to a polymicrobial infection of the perineal, genital, or perianal region. It is characterized by rapid tissue destruction and systemic signs of toxicity. It is more frequent in males and immunocompromised patients, such as patients with poorly controlled diabetes, alcoholism, or human immunodeficiency virus (HIV) infection. Treatment often involves surgical intervention, broad-spectrum antibiotic therapy, fecal diversion surgery, and negative pressure wound therapy (NPWT). Delays in diagnosis are associated with high mortality due to rapid progression to septic shock.

## Introduction

Fournier gangrene is a type of necrotizing soft tissue infection (NSTI). NSTIs are divided into monomicrobial and polymicrobial infections and can be present in one or multiple layers of the soft tissue: cellulitis (infection involving the inner layers of the skin), fasciitis (infection of the fascia of a muscle), and myositis (a rare NSTI involving the muscle). Early manifestations may be subtle, and the diagnosis is typically achieved when there are already severe symptoms [1].

The origin of the infection is not always clear, but common identifiable sources of infection may include the skin and urinary and gastrointestinal tract (e.g., perforated colorectal cancer, Crohn’s disease, or diverticulitis) [2].

Current treatments include intensive surgical debridement, systemic antibiotic administration, fecal diversion, and negative pressure wound therapy (NPWT) [3]. The need and timing to perform some kind of fecal diversion are still in debate [4].

Herein, we report a case of successful Fournier gangrene management without the need for colostomy. Treatment involved surgical debridement, antibiotic therapy, and NPWT. We obtained the patient’s consent for the publication of the case, as well as the use of images.

## Case presentation

A 75-year-old male with a history of arterial hypertension and prostatic cancer, without previous surgeries, came to the emergency department with testicular pain for six days, which aggravated in the last two days. He noticed a perianal tumefaction 15 days before the beginning of the pain. By then, he was treated by the primary care physician with antibiotic and anti-inflammatory drugs due to a small perianal abscess with spontaneous drainage. Since then, the patient noticed testicular enlargement and pain, which irradiated to the right side of the abdomen. The patient denied fever and gastrointestinal or urinary symptoms.

**Figure 1:**
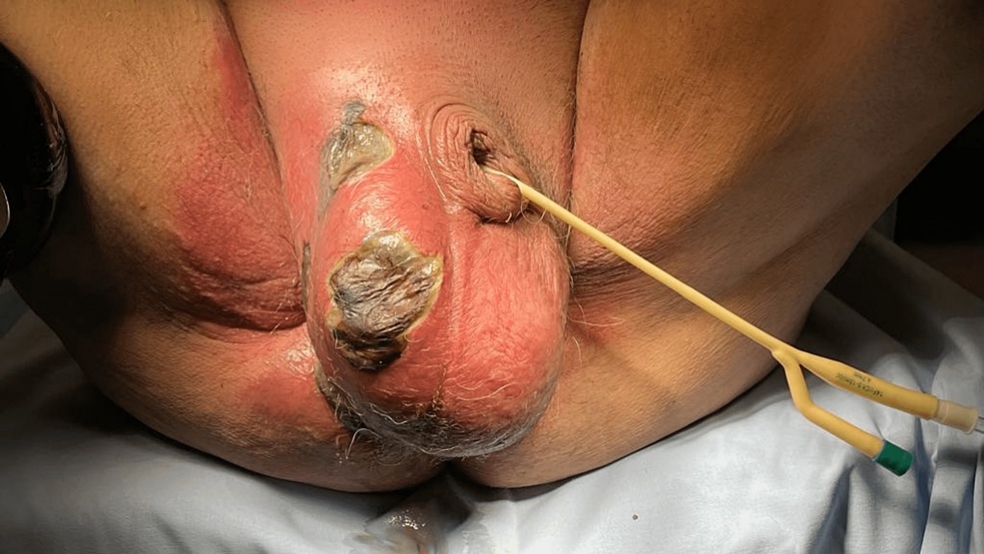
Inflammatory signs of the scrotum with extension to the right iliac fossa and skin necrosis of the scrotum (photo before the first surgical debridement).

On physical examination, he was hemodynamically stable (blood pressure of 123/65 mmHg and heart rate of 82 bpm) and afebrile, and he presented inflammatory signs, abscess, and skin necrosis of the scrotum with extension to the right iliac fossa (Figure 1).

Analytically, he had increased inflammatory parameters (leukocytosis of 14.70 × 10³/µL (reference range: 4-11 × 10³/µL) and elevated C-reactive protein at 21.03 mg/dL (reference value: <0.5 mg/dL)), and scrotum ultrasound revealed multiple gas foci and thickening of the scrotum.

**Figure 2:**
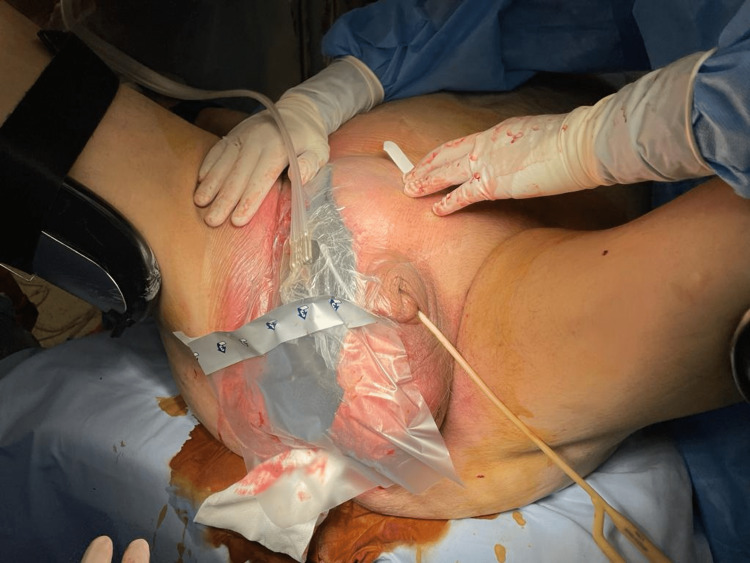
Placement of NPWT after the first surgical debridement. NPWT: negative pressure wound therapy

The patient underwent surgical debridement of the necrotic areas of the scrotum, right perianal region, and right iliac fossa followed by placement of NPWT (100 mmHg) (Figure 2). On the third postoperative day, the patient presented with hypotension, urinary output decrease, and recrudescence of cutaneous inflammatory signs in the right inguinal area. Therefore, he was again subjected to surgical debridement with widening of the dissection to the abdominal right flank and application of a new NPWT.

*Escherichia coli* was isolated in the pus culture, which was sensitive to piperacillin/tazobactam, and blood cultures were negative. A 14-day cycle of piperacillin/tazobactam was completed.

The patient was submitted to five more wound dressings in the operating room at intervals of three days. The negative pressure applied varied from 100 to 120 mmHg.

In the last surgical wound dressing, NPWT was replaced by a suction drain. Attempts to divert feces (placing a Pezzer tube) were not successful due to fecal consistency, and anal plugs were not available.

By the time of hospital discharge (day 32), the wound was clean without fecal contamination. Follow-up appointments revealed a positive evolution with healing of the inguinal region skin and a small area of dehiscence in the scrotum skin without inflammatory signs.

## Discussion

Surgical debridement, systemic antibiotic administration, fecal diversion, and negative pressure wound therapy are the cornerstones of Fournier gangrene treatment [4,5].

It is accepted that the most significant modifiable risk factor associated with NSTI mortality is the delay of surgical intervention. Therefore, early and aggressive surgical debridement is indispensable in the control of disease progression [1]. Prompt surgery allows the lesions to be opened, reduces tension, and eliminates the necrotic tissues. Repeated debridement is warranted whenever clinical or imaging deterioration (ultrasonography or CT) is present since it is the only definitive care [1,4].

The need for fecal diversion remains controversial. Undoubtedly, a fecal diversion must be performed whenever there is sphincter commitment or poor wound healing or when it is not possible to perform periodical surgical revisions. A colostomy is the most common procedure for fecal diversion because it allows the elimination of solid and formed stools that lead to less contamination of the surrounding skin. Its potential benefits include the reduction of wound contamination and the possibility of early enteral nutrition, which allows rapid improvement of nutritional status and, therefore, facilitates wound healing [4]. On the other hand, it requires an abdominal surgical procedure to perform the colostomy followed by a second procedure to reconstruct with all the pitfalls associated and also the costs of colostomy material [3,5]. In our case, although the origin of the disease was a perianal abscess and extensive perianal debridement was performed, fecal contamination was avoided mainly due to frequent wound dressings and careful placement of NPWT.

An alternative method to colostomy is a bowel management catheter such as anal plugs. However, it can only be used in cases where the anal sphincter is not impaired. Thereby, colostomy should be reserved for cases with sphincter injury [4].

NPWT is a wound closure method useful in infected wounds (acute or chronic). Studies have shown that the most effective negative pressure value is 125 mmHg [3]. The benefits of NPWT are well-documented for Fournier gangrene [6]. The negative pressure allows excess exudate to be absorbed, which facilitates the wound healing process by reducing edema and increasing blood supply. The recovery of tissue oxygenation stimulates neovascularization and increases cellular proliferation at the periphery of the wound, thereby reducing bacterial load. These changes in the affected tissue lead to the downsizing of the wound dimensions due to the contraction of the circumference of the wound [6].

However, the complex anatomy of the perineum remains a challenge in maintaining the airtight seal necessary to keep the negative pressure over the affected area [3,6].

## Conclusions

Nowadays, in most centers, surgeons seek to avoid performing colostomy, unless it is an absolute imperative. In this case report, a successful case of Fournier gangrene in which colostomy was not performed gives strength in managing patients with surgical debridement and negative pressure wound therapy. The importance of having differentiated healthcare providers who are comfortable with the use of negative pressure wound therapy and its limitations (e.g., air leaks that lead to prolonged healing time and increase the number of surgical procedures) are highlighted in the present case.
